# Effects of a transformer-based AI-based application to support incontinence-associated dermatitis and pressure injury assessment, nursing care and documentation: Controlled pilot intervention study

**DOI:** 10.1016/j.ijnsa.2026.100479

**Published:** 2026-01-07

**Authors:** Hannah Pinnekamp, Vanessa Priester, Johanna Steidle, Khalid Majjouti, Alexander Brehmer, Michaela Tapp-Herrenbrück, Michael Aleithe, Jens Kleesiek, Bernadette Hosters, Uli Fischer

**Affiliations:** a LMU Hospital Munich, Nursing Department, Department of clinical nursing research and quality management, Munich, Germany; b University Hospital Essen, Department Nursing Development and Nursing Research, Essen, Germany; c University Hospital Essen, Institute for Artificial Intelligence in Medicine (IKIM), Essen, Germany; d sciendis GmbH, Leipzig, Germany; e Catholic University of Applied Sciences Munich (KSH), Munich, Germany

**Keywords:** Nursing process, Artificial intelligence, Pressure Injury, Irritant dermatitis, Fecal incontinence, Urinary incontinence, Workload, Guideline adherence

## Abstract

**Background:**

Artificial intelligence (AI) is playing an increasingly important role in nursing care, including wound management. Differentiating pressure injuries from incontinence-associated dermatitis is clinically challenging, often leading to misclassification. Although AI-based wound assessment is advancing, few models specifically address incontinence-associated dermatitis, and clinical evidence remains limited. The KIADEKU project developed and piloted a transformer-based AI app to support care for these wounds.

**Objective:**

The aim of this pilot intervention study was to assess the impact of the AI-based app on duration of wound assessment, dressing changes, documentation, nursing staff task load, and guideline adherence. Secondary aims included evaluating the AI’s accuracy and app usability compared to standard systems.

**Design:**

This monocentric, non-randomized controlled study was conducted in two sequential phases: a control phase with conventional wound management, followed by an intervention phase utilizing the AI-based app.

**Setting and Participants:**

The study included 88 voluntary nurses caring for pressure injuries and incontinence-associated dermatitis in adult patients on seven participating wards of LMU Hospital.

**Methods:**

Wound care was systematically observed, and nurses completed questionnaires on task load, usability, and covariates. Outcomes were measured using standardized protocols, validated tools (NASA Task Load Index (NASA-TLX), Usability Metric for User Experience (UMUX-LITE)) and expert-defined indicators. Statistical analyses included descriptive statistics, group comparisons (t-test, Mann-Whitney U test), and multivariate linear regression adjusting for covariates. An independent wound assessment validated AI-generated predictions, with accuracy evaluated using F1-scores.

**Results:**

A total of 88 wound care sessions were analysed. The intervention group had a statistically significantly longer mean duration of care and documentation (12.84 vs. 9.20 min; p = 0.002; 95 % CI: –5.59; –1.41 min) and higher guideline adherence (mean rank = 50.91 vs. 38.38; p = 0.017). Nurse task load showed no statistically significant group differences. Regression analysis identified AI app use, nurse qualification, and wound severity as statistically significant predictors of care duration, while AI use did not predict task load or guideline adherence. Usability ratings were similar to standard systems. Model performance showed high accuracy in identifying wound types, but lower accuracy in classifying their categories.

**Conclusions:**

This pilot study is the first to evaluate an AI-based app supporting nursing wound management for pressure injuries and incontinence-associated dermatitis. While the app did not reduce care duration or nurse workload, it may have potential to improve guideline adherence. Limitations included limited user experience and sample bias. Future multicentre studies with larger samples and randomized trials are needed to validate findings and support clinical integration.

**Registration:**

www.drks.de DRKS00031355. Registered 05/04/2023, first recruitment 31/05/2023.

**What is already known**

• Differentiating pressure injuries from incontinence-associated dermatitis is clinically challenging, often resulting in misclassification.
• AI-based wound assessment tools are emerging, but few specifically address incontinence-associated dermatitis, and clinical evidence on their effectiveness is limited.

**What this paper adds**

• The AI-based wound management app did not reduce wound care duration or nurse task load but was associated with improved guideline adherence.
• The AI model showed high accuracy in distinguishing wound types, although classification of specific categories was less precise.

## 1 Introduction

### 1.1 Background

AI models are increasingly being developed and piloted in nursing care, including wound care. These technologies hold significant potential for standardizing wound imaging, enabling accurate wound measurements, identifying complications, supporting classification and treatment decisions, and improving documentation efficiency (Dabas et al., 2023; Lau et al., 2022; Mohammed et al., 2022).

Such AI systems are trained on large datasets to autonomously detect patterns in wound data and make predictions or decisions. Various algorithms and network architectures are employed and often combined to optimize performance.

In clinical nursing wound care, differentiating between pressure injuries and incontinence-associated dermatitis is particularly challenging, especially in the early stages, as both conditions may present with similar visual characteristics (Beeckman et al., 2011). Pressure injury result from prolonged pressure exposure and can range from non-blanchable erythema of intact skin to full-thickness tissue loss involving subcutaneous fat, muscle, and even bone (European Pressure Ulcer Advisory Panel; National Pressure Injury Advisory Panel, 2019). Without timely and effective prevention and intervention, pressure injuries can lead to negative patient outcomes and increased healthcare costs due to extended hospital stays (Labeau et al., 2021).

In contrast, incontinence-associated dermatitis is caused by prolonged exposure to urine or faeces, resulting in inflammation of the skin. It presents as erythema and may involve skin loss or infection. Incontinence-associated dermatitis increases the risk of pressure injuries and secondary infections (Gray et al., 2007). It is a highly prevalent condition among patients with incontinence. Prevalence in hospitalized patients ranges from 1.44 % to 23 % among hospitalized patients (Gray und Giuliano 2018; Wei et al., 2020; Arnold-Long und Johnson 2019), reaching 36.2 % in older patients (Ferreira 2020) and up to 45.7 % in patients with incontinence (Gray und Giuliano, 2018). Patients with incontinence-associated dermatitis also show longer hospital stays, higher readmission rates, and increased costs (Kayser et al., 2021).

Due to low awareness of incontinence-associated dermatitis and the diagnostic challenges described above, misclassification is common, often resulting in inappropriate or delayed treatment (LeBlanc et al., 2016; Beeckman, 2017). Studies further indicate that nurses face challenges and knowledge gaps in the correct classification, prevention, and management of incontinence-associated dermatitis (Liu et al., 2023; Barakat-Johnson et al., 2022; Kılıç et al., 2024). However, knowledge, attitudes, social norms, and perceived behavioral control significantly influence incontinence-associated dermatitis prevention (Asiri et al., 2024), and therefore represent important factors to consider in efforts to improve care in this area. Additionally, underreporting of incontinence-associated dermatitis remains an issue, due to the absence of a dedicated International Classification of Diseases (ICD-10) code and the limited implementation of standardized classification tools such as the Ghent Global Incontinence-Associated Dermatitis (IAD) Categorization Tool (GLOBIAD) (Beeckman et al., 2017; Kayser et al., 2021).

Beyond the differentiation between pressure injuries and incontinence-associated dermatitis, general challenges in wound documentation include incomplete records of relevant wound characteristics, inaccurate measurements due to irregular wound shapes or locations, and poorly defined wound margins. Low-tech approaches further reduce interobserver reliability. (Do et al., 2021; Keast et al., 2004; Haghpanah et al., 2006; Wang et al., 2017)

Moreover, documentation is time-consuming, especially in facilities like LMU Hospital, where it still relies on digital cameras and paper rulers, followed by manual transfer into electronic documentation systems.

AI-based solutions could help address both the differentiation of wound types and the documentation burden. While several models have already been developed and piloted for pressure injury management (Jiang et al., 2021; Alves et al., 2025), to our knowledge, no AI models currently incorporate incontinence-associated dermatitis. Additionally, research on the clinical impact of such tools on direct nursing care remains scarce.

Key barriers to clinical implementation of AI include limited technical and informatics skills among clinical staff, as well as ethical concerns (Abuzaid et al., 2022; Gerich et al., 2022). Therefore, active involvement of nurses as end users in the research and development process is essential to ensure that their needs are adequately addressed (Gerich et al., 2022; Zhou et al., 2021). This involvement can enhance the utility of AI for nurses and, ultimately, improve patient outcomes.

To address these issues, the KIADEKU (KI^1^-IAD-DEKUbitus^2^) project (German Clinical Trials Register: DRKS00029961) developed a transformer-based AI model designed to support nurses in assessing pressure injuries and incontinence-associated dermatitis, facilitate documentation, and promote the implementation of personalized, evidence-based nursing interventions.

The project comprises three components: (1) a technical sub-study on AI development (Brehmer et al., 2025), (2) a mixed-methods sub-study involving nurses in the co-development process (Majjouti et al., 2025), and (3) the clinical interventional study described here, in which the AI-based app was piloted in clinical care.

To develop the AI model, a literature-based minimal dataset of relevant wound characteristics was created and refined through consensus with nurses (Majjouti et al., 2025). A total of 1,555 wound images from the hospital information systems of two university hospitals were included. These images were pre-screened according to inclusion and exclusion criteria, anonymized, validated, segmented, and annotated by the study team. Using these images, a transformer-based AI model was trained, achieving an F1-score of 93.23 % for the binary classification of pressure injuries versus incontinence-associated dermatitis, and 75.43 % for pressure injury classification and 53.20 % for incontinence-associated dermatitis classification (Brehmer et al., 2025). The model was integrated into the wound management app Wundera® by project partner sciendis GmbH and piloted in clinical nursing practice. The aim of this non-randomized controlled pilot intervention study was to quantitatively assess the impact of the AI-based app on nursing care for pressure injury and incontinence-associated dermatitis.

### 1.2 Objectives

The primary objective of the study was to examine the impact of using the AI-based app on the following dependent variables: duration of wound assessment, dressing changes, and documentation, the task load of the nursing staff, and guideline adherence. Additionally, we adjusted for potential covariates such as wound severity and nurse qualification. We hypothesized that the use of the AI-based app would reduce the duration of care and documentation, decrease nursing task load and enhance guideline adherence.

The secondary objective was to evaluate the predictive performance of the AI model in clinical practice and assess the usability of the AI-based app compared to the standard documentation system.

## 2 Methods

### 2.1 Design

This monocentric non-randomized controlled pilot intervention study comprised two consecutive data collection phases. In the first phase (control), conventional wound care and documentation of pressure injury and incontinence-associated dermatitis were observed using a standardized protocol, and the responsible nursing staff completed a questionnaire.

In the second phase (intervention), following staff training, the AI-based app was piloted in clinical practice. Observations and surveys were repeated following the same procedures as in the control phase. Additionally, an on-site study team member performed an independent wound assessment, which served as ground truth for evaluating the AI model’s performance in clinical care. Due to the nature of the intervention, neither participants nor observers could be blinded. No changes to the study methods, including eligibility criteria or procedures, were made after trial commencement.

### 2.2 Setting

The study was conducted at LMU Hospital, which offers comprehensive services across all medical specialties and has a capacity of over 2,000 beds. For this study, we selected four intensive care units, one intermediate care unit, and two general wards that had a particularly high incidence of pressure injuries due to the patient population treated. Additionally, it was ensured that the selected wards had adequate Wi-Fi coverage to guarantee the smooth operation of the AI-based app. The wards differed in medical specialty, number of beds, and number of staff members. The nursing managers and ward supervisors approved the participation of their respective wards. Furthermore, consent was obtained from the staff council, the data protection officer, and the ethics committee of the Medical Faculty of LMU.

### 2.3 Participants

We included voluntary nursing professionals who performed wound care for pressure injury and incontinence-associated dermatitis in adult patients on the selected wards. Both newly developed wounds and pre-existing wounds in newly admitted patients were included. Since incontinence-associated dermatitis occurs only in the pelvic region, only wounds in this area were included. Other types of wounds were excluded, as were minors, patients too unstable for repositioning during dressing changes, patients for whom life-prolonging measures had been discontinued, or those assessed by nursing staff to be in the acute dying process. To ensure that no other wound types were included, a study team member on site verified the wound type, and data collection was conducted only if it met the inclusion criteria. Since the AI was trained for the binary distinction between pressure ulcers and incontinence-associated dermatitis, only cases in which both wound types were sufficiently spatially separated to allow photographing and managing a single wound type were included, and cases where this was not possible were excluded. All nursing professionals were required to speak fluent German and to sign informed consent forms before inclusion in the study. Nursing staff who did not provide informed consent for observation and survey participation, who were not fluent in German, or who were unlicensed caregivers, trainees, apprentices, or other professional groups performing wound care were excluded.

The inclusion and exclusion criteria for the data used in AI training can be found in the paper on technical development (Brehmer et al., 2025).

The input data for AI prediction included wound photographs taken via the app as well as additional patient data entered by the nursing staff (immobility, incontinence status, and sensibility assessments).

### 2.4 Standard care

In the control phase, conventional wound care was provided based on the clinic’s standard operating procedures, which are based on the German Network for Quality Development in Nursing (DNQP) expert standards 'Pressure Injury Prevention in Nursing and Nursing Care for Patients with Chronic Wounds' (Büscher et al., 2021; Schiemann, 2009) and are provided via an internal digital platform. For digital wound documentation, nurses captured wound images using a digital camera and a paper reference ruler, which were then manually transferred to the computer. The images were uploaded to the patient’s electronic health record and supplemented with additional details on wound characteristics and care provided. During this phase, wound care procedures were systematically observed, and the responsible nursing staff completed questionnaires regarding task load and related covariates. No changes were made to the infrastructure or processes during the study period.

### 2.5 Intervention

At the beginning of the intervention phase, participating wards received training on app usage, interpretation of AI predictions, and associated risks. Nurses were also able to test the app prior to data collection.

The intervention consisted of using the AI-based app (version 1.0) for wound assessment and documentation on tablets or smartphones. During wound care, nurses entered basic patient information (mobility, incontinence, sensitivity) and captured a wound photograph. These inputs were mandatory in the documentation process, thereby preventing missing input data for the AI model. The wound edge was delineated manually and automatically measured using a calibration patch, the margins of which were previously marked manually. In cases of incontinence-associated dermatitis classified as 1A and 1B, as well as stage 1 pressure injuries, the border of the erythema was defined as the wound margin. The app displayed predicted wound type, severity, and other characteristics, along with an accuracy indicator. Predictions below predefined thresholds (80 % for wound type, 60 % for classification) were not shown. All entries required manual review and confirmation.

Subsequently, the app suggested evidence-based nursing interventions and additional information (e.g., wound categories, contraindications) based on the documented wound characteristics, building on prior work (Majjouti et al., 2025). Final documentation of care and additional measures was completed within the app.

Compared to conventional care, the intervention replaced camera-based image capture and delayed PC documentation with bedside digital documentation and AI-supported pre-filling of wound characteristics, aiming to reduce documentation time and nursing task load. Additionally, supplementary information fields and evidence-based recommendations were integrated to further support guideline adherence.

### 2.6 Outcomes

The primary study outcomes included the duration of wound assessment, wound care, and documentation, nursing staff task load, and guideline adherence. Relevant covariates, such as overall workload, wound severity, professional qualification and experience, ward type, and nurse-to-patient ratio, were collected to control for potential effects.

The duration of wound care and documentation was recorded using a standardized observation protocol with clearly defined start and end points. The start was defined as the removal of the wound dressing. If no dressing was present, the first hands-on activity after patient positioning marked the start. Preparatory tasks and interruptions were excluded. Documentation performed separately from the care episode was recorded and added to total care time. Documentation time was measured from opening the digital wound documentation system or connecting a digital camera until saving the entry. It was also noted if a wound photograph was taken and whether previous values were reused.

To assess guideline adherence, five indicators were selected based on the 'Prevention and Treatment of Pressure Ulcers' guideline (European Pressure Ulcer Advisory Panel; National Pressure Injury Advisory Panel, 2019) and, due to the lack of specific guidelines for incontinence-associated dermatitis, on recommendations from current literature on incontinence-associated dermatitis (Beele et al., 2018; van Wissen et al., 2019). The indicators were chosen to be generally applicable to both wound types, as well as suitable for distinguishing between the two wound types. These indicators were developed during an expert workshop and subsequently validated via Delphi consensus with clinical experts in the KIADEKU mixed-methods sub-study. The indicators included:

• performing a blanch test in the presence of erythema (recommendation 9.1) to differentiate a pressure injury from other types of erythema
• accurate assessment of wound type and classification (recommendation 9.2)
• applying wound edge protection in the presence of maceration at the wound edge or surrounding skin (recommendation 3.1)
• ensuring pressure relief for pressure injuries (recommendations 5.1 and 5.5)

To determine the accuracy of the nurse’s assessment of wound type and classification, it was compared with the independent evaluation of a study team member on site. Each indicator was scored dichotomously: 'No' (0) and 'Yes or not required' (1). A sum score ranging from 0 to 5 was calculated, with higher scores reflecting better guideline adherence.

The observation protocol also recorded the ward type: general wards (1), intermediate care units (2), and intensive care units (3). To evaluate the accuracy of AI predictions for wound type and classification, an independent assessment was performed by a trained study team and documented as the ground truth in the observation protocol.

Additional data were collected via a post-care nurse questionnaire, including age, gender, perceived workload during the shift, nurse-to-patient ratio, years of professional experience, highest qualification, and specialized training.

Task load during wound assessment, care, and documentation was measured using the validated NASA Task Load Index (NASA-TLX), which consists of 6 items rated on a 21-point scale (ranging from 0 to 100 in 5-point increments). The final score was calculated using the standard NASA-TLX scoring formula, yielding values between 0 and 100, with higher values indicating greater task load (Human Performance Research Group, 2023).

Overall workload during the shift was measured using a Likert scale (1 = 'Considerably lower than average' to 5 = 'considerably higher than average'). Nurses were also asked to report the number of patients they cared for during the observed shift.

Professional qualification was categorized according to the German Qualifications Framework:

• three-year nursing training (Registered Nurse) (1)
• specialist non-academic training certified by the German Hospital Federation (DKG) (2)
• bachelor’s degree (3)
• master’s degree (4)

Additional relevant certifications (e.g., wound care expert) were also recorded.

Usability, defined as a secondary study outcome, was assessed using the UMUX-LITE questionnaire due to its brevity and comparability (Borsci et al. 2020). Nurses completed it for the standard clinical system (control) or the AI-based application (intervention) and the score and weighted score were calculated (Lewis et al., 2013). In addition, the F1-score for the predictive performance of the AI model in clinical practice was evaluated as a further secondary outcome, based on a direct comparison between the AI-generated classifications and the wound assessments conducted by the study team member at the point of care (Powers, 2011).

No changes were made to the study outcomes after the commencement of the study.

### 2.7 Sample size

The a priori sample size calculation was conducted based on the linear regression analyses, which were the primary statistical methods planned to address the main research hypotheses. Group comparisons were performed as supplementary analyses to further investigate differences between groups. Due to the pilot nature of the study and the limited sample size, no separate a priori sample size calculations were conducted for these supplementary analyses.

The sample size calculation was conducted using G*Power software (Faul et al., 2007). The calculation assumed medium effect sizes according to Cohen (1988), with an alpha level of 0.05 and a statistical power of 0.80. To examine the relationships between the independent variable (use of the AI-based app), up to three potential covariates, and the dependent variables (duration, task load, guideline adherence) using multivariate linear regression models, a sample size of 85 nursing professionals was determined to be necessary. To account for an anticipated dropout and missing data rate, a total of 93 nursing professionals were recruited.

### 2.8 Enrolment

Eligible cases were identified by searching wound documentation systems and through direct contact between the study team and the hospital wards. If wounds meeting the inclusion and exclusion criteria were found, the study team contacted the ward to schedule an appointment for the next wound care session. On the agreed date, a member of the study team informed the responsible nurse about the study. If the nurse declined participation, no observation or data collection took place. However, the patient could still be included for a subsequent wound care session if a different nurse agreed to participate. If the nurse provided written informed consent, the study team conducted an observation and the nurse completed a questionnaire on a tablet after care was provided.

The study primarily targeted newly occurring wounds or newly admitted patients with pre-existing wounds. Due to organizational factors, delays in inclusion sometimes occurred, such as admissions during weekends, nurses opting out of participation, or delays in wound documentation. Each patient was included in the study only once.

To encourage participation, local wound care experts were involved in the development of the AI-based app as part of the mixed-methods sub-study. Additionally, participating nurses received a voucher as compensation for the additional effort associated with the study.

After careful consideration and consultation with the ethics committee, informed consent from patients was deemed unnecessary because wound care provided during the study adhered to standard procedures and only differed in the documentation system used. Furthermore, no data were collected that could identify individual patients. Nevertheless, patients were informed about the study prior to wound care.

### 2.9 Data collection and management

Wound care observations were conducted by trained nursing scientists using a digital observation protocol on tablets, implemented via Lime Survey (Limesurvey GmbH, 2024). All collected data were anonymized. To ensure that wound documentation could be reliably linked with observation and survey data, while maintaining strict data privacy and security, an anonymized ID was generated within the AI-based app and recorded on the observation form. Neither this ID nor any data collected in the wound documentation, observations, or questionnaires could be used to identify patients or nurses.

All data, signed consent forms and voucher receipt confirmations were securely stored in a password-protected folder accessible only to the study team. Upon completion of data collection, data were automatically exported and statistically analysed using SPSS Version 28.0.0.0 (IBM Corp, 2021).

Due to the relatively small sample size, the limited complexity of the study, and the clearly defined endpoints, a data monitoring committee was not deemed necessary.

### 2.10 Statistical methods

The included cases were described according to ward type, wound type and severity, and characteristics of the nursing staff. Metric dependent variables with an approximately normal distribution were reported as mean and standard deviation (SD), and group differences with homogeneity of variance were assessed using the t-test. Categorical data and non-normally distributed variables were reported as median and interquartile range (Q1-Q3), and group differences were analysed using the Mann–Whitney U test. Effect sizes were reported for both tests (Cohen’s d, Pearson correlation coefficient r), and confidence intervals and post-hoc power were calculated for the t-test. Statistical significance was defined as two-tailed p < 0.05, with power set at 0.80. Boxplots and frequency tables were used for visualization.

To explore associations between the use of the AI-based app and the dependent variables, stepwise multiple linear regression analyses were conducted (entry criterion: *p* ≤ 0.05; removal criterion: *p* ≥ 0.10). Potential covariates such as wound severity, nursing staff’s professional experience and nurse qualification, ward type, nurse-to-patient ratio, and overall shift workload were included where relevant. Model assumptions were assessed via inspection of scatterplots (linearity of relationships), Durbin–Watson statistic (residual independence), variance inflation factors (VIF) (multicollinearity), Shapiro–Wilk tests and histogram inspection (normality of residuals), and the modified Breusch–Pagan test (homoscedasticity). Model fit was evaluated using adjusted R² and F-statistics. Due to violations of the normality and homoscedasticity assumptions, non-parametric bootstrapping with 1,000 resamples (bias-corrected and accelerated (BCa) 95 % confidence intervals (CIs)) was used for robust estimates. Missing data were handled via listwise deletion. No Bonferroni correction was applied, as each model tested a specific hypothesis within this exploratory pilot study.

Usability of the documentation systems was assessed using the weighted UMUX-LITE score and analysed descriptively. Group differences were tested using the Mann–Whitney U test due to variance heterogeneity.

The F1-score, defined as the harmonic mean of precision and recall, was used to evaluate the accuracy of wound type and severity classification by the AI-based app. The ground truth was defined as the assessment made by the study team member at the point of care. Precision quantifies the proportion of true positive predictions among all predicted positives, while recall represents the proportion of true positives correctly identified among all actual positives (Powers, 2011).

The F1-score was calculated for each class individually, and the micro-F1 score, computed by aggregating true positives, false positives, and false negatives across all classes before calculating precision and recall, was used to assess overall performance. This metric is particularly suitable for imbalanced class distributions.

All analyses were performed using IBM SPSS Statistics (Version 29.0.0.0) and G*Power software (Faul et al., 2007).

### 2.11 Registration

German Clinical Trials Register (DRKS) (DRKS00031355). Registered on April 5th, 2023.

### 2.12 Protocol

The study protocol was published open access (Pinnekamp et al., 2025).

### 2.13 Ethics approval

The study has received approval from the Ethics Committee of the LMU Medical Faculty (process number 22-0970), the Data Protection Officer of LMU Hospital (process number 1952) and the Staff Council of LMU Hospital. All procedures were conducted in accordance with the Declaration of Helsinki, and no significant changes to the study protocol have been made. We ensured compliance with all Ethics Committee requirements and obtained informed consent from all participants prior to enrolment. Adjustments to the study protocol that became necessary during the study, were updated accordingly in the study registration.

## 3 Results

Data collection for the control group began in May 2023 and continued until January 2024, when the required sample size was reached. After completion of the AI app development and nurse training, data collection for the intervention group started in July 2024 and was completed in April 2025 upon reaching the target sample.

A total of 93 wound care sessions were observed, followed by questionnaires completed by the nursing staff. Of these, 88 complete datasets were included in the analysis. Five datasets were excluded due to incomplete questionnaires or wound care being performed by non-nursing staff. In 45 cases, wound care was performed conventionally without the AI-based app (control), and in 43 cases with the AI-based app (intervention). Data collection was conducted in 15 cases on general wards, 2 cases on intermediate care units, and 71 cases in intensive care units. The observed wound care procedures according to wound types, i.e., pressure injury, incontinence-associated dermatitis, or the presence of both wound types, and their corresponding classifications are presented in Table 1.

**Table 1:** Number of observations by wound type and classification.

		**Incontinence-associated dermatitis** (GLOBIAD^1^)					**Total**
		**None**	**1A**	**1B**	**2A**	**2B**	
**Pressure Injury** (EPUAP^2^)	**None**	0	5	0	7	1	13
	**Stage 1**	4	0	0	0	0	4
	**Stage 2**	37	1	2	0	1	41
	**Stage 3**	9	0	0	0	0	9
	**Stage 4**	2	0	0	0	1	3
	**Deep Tissue Injury**	13	0	0	1	0	14
	**Unstageable Pressure Injury**	4	0	0	0	0	4
**Total**		69	6	2	8	3	88

1 Ghent Global IAD Categorization Tool

2 European Pressure Ulcer Advisory Panel

The characteristics of included nurses are presented in Table 2.

**Table 2:** Characteristics of included nurses.

Characteristics of nurses		Control group	Intervention group
Gender	Female	25	28
	Male	20	15
Age group	20-29	16	15
	30-39	16	13
	40-49	3	8
	50-59	9	6
	≥ 60	1	1
Qualification	Three-year nursing training (Registered Nurse)	16	19
	DKG^1^-recognized specialized further training	14	15
	Bachelor’s degree	13	9
	Master’s degree	2	0
Other relevant qualifications	Wound expert certification	7	3
Total		45	43

1 German Hospital Federation

The nursing staff had between 1 and 45 years of professional experience (mean 13.47 (SD 10.85)) and cared for between 1 and 13 patients per shift (mean 3.23 (SD 3.30)).

No harms or unintended effects were observed in any of the study groups. Furthermore, there were no reported performance issues related to the AI-based app throughout the study period.

### 3.1 Descriptive results and group comparisons of dependent variables

**Fig. 1:**
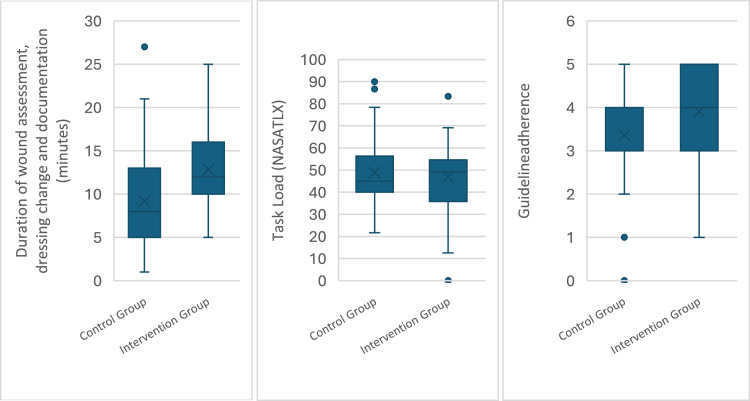
Box Plots of dependent variables in control and intervention groups.

The mean duration of care and documentation in the control group was 9.19 minutes (SD 5.93), and in the intervention group it was 12.84 minutes (SD 4.49) (see Fig. 1). After confirming t-test assumptions, an independent two-tailed t-test revealed statistically significant group differences, with a mean difference of -3.65 minutes (95 % CI [-5.89, -1.41]), t (86) = -3.24, p = 0.002, and statistical power of 0.87. The effect size was in the medium-to-large range (Cohen’s d = 0.69, 95 % CI [-1.12, -0.26]).

The mean task load (NASA-TLX scale from 0 to 100) in the control group was 48.83 (SD 14.36), and in the intervention group it was 46.91 (SD 15.09) (see Fig. 1). After confirming t-test assumptions, an independent two-tailed t-test revealed no statistically significant group differences, with a mean difference of 1.92 (95 % CI [-4.39, 8.24]), t (84) = 0.61, p = 0.546, and statistical power of 0.09. The effect size was small (Cohen’s d = 0.13, 95 % CI [-2.93, 0.55]).

The mean score for guideline adherence (scale from 0 to 5) in the control group was 3.36 (median = 3; Q1–Q3 = 3–4), and in the intervention group it was 3.91 (median = 4; Q1–Q3 = 3–5) (see Fig. 1). The Mann-Whitney U test showed a statistically significant difference between the groups (U = 692.00, p = 0.017, r = 0.26), with the control group having a mean rank of 38.38 and the intervention group a mean rank of 50.91. The Hodges-Lehmann median difference was 1.0 (95 % CI [0.0, 1.0]), indicating a possible median difference. Because the confidence interval includes zero, there is uncertainty regarding the estimate.

Descriptive differences of the items used for evaluating guideline adherence in the control group and in the intervention group are presented in Table 3.

**Table 3:** Descriptive results of the guideline adherence items in the control and intervention groups.

Guideline adherence items			Control Group	Intervention Group
Was the blanch test performed in the presence of erythema?	Yes, or not required	n	28	38
		%	62,2 %	88,4 %
	No, although indicated	n	17	5
		%	37,8 %	11,6 %
Was pressure relief implemented in the presence of a pressure injury?	Yes, or not required	n	30	33
		%	66,7 %	76,7 %
	No, although indicated	n	15	10
		%	33,3 %	23,3 %
Was skin protection implemented in the presence of moist skin?	Yes, or not required	n	39	34
		%	86,7 %	79,1 %
	No, although indicated	n	6	9
		%	13,3 %	20,9 %
Correct assessment of the wound type		n	41	38
		%	91.1 %	88.4 %
	Correct assessment of wound classification	n	13	25
		%	31.7 %	65.8 %
	Incorrect assessment of wound classification	n	28	13
		%	68.3 %	34.2 %
Incorrect assessment of the wound type		n	4	5
		%	8.9 %	11.6 %
Total			45	43

The Mann–Whitney U test revealed a statistically significant group difference in blanch test performance (p = 0.005; mean rank control = 38.88, intervention = 50.38; r = 0.30) and in wound classification following correct wound type determination (*p* = 0.009; mean rank control = 35.80, intervention = 47.78; r = 0.29). However, no Hodges-Lehmann median difference could be determined for blanch test performance (95 % CI [0, 0]) or for wound classification after correct wound type determination (95 % CI [0, 1]). No statistically significant group differences were found for pressure relief on existing pressure injuries (*p* = 0.297; mean rank control = 42.33, intervention = 46.77; r = 0.11), skin protection on moist skin (*p* = 0.346; mean rank control = 46.13, intervention = 42.79; r = 0.10), or correct assessment of wound type (*p* = 0.673; mean rank control = 45.09, intervention = 43.88; *r* = 0.04).

### 3.2 Linear regression models

#### 3.2.1 Model for duration of wound assessment, dressing change and documentation

For the regression model predicting the dependent variable duration of care and documentation, the inclusion of the variables use of AI-based app, nurse qualification, and wound severity yielded an adjusted R² of 0.237, with an F-value of 9.983 (*p* < 0.001) and power of 0.995.

The regression equation is as follows:

Y=−1.826+3.921×(UseofAI−basedapp+1.966×(Nursequalification)+4.756×(WoundSeverity)

The standardized regression coefficients were 0.355 (p < 0.001, 95 % CI [0.166, 0.544]) for use of AI-based app, 0.304 (p = 0.002, 95 % CI [0.116, 0.492]) for nurse qualification, and 0.245 (p = 0.011, 95 % CI [0.058, 0.432]) for wound severity.

These findings indicate that, contrary to the initial hypothesis, the use of the AI-based app was associated with an increase in the duration of wound care and documentation.

The independence of residuals was confirmed, and multicollinearity among predictors was ruled out. Since the assumption of homoscedasticity was violated, bootstrapping was used, revealing statistically significant positive effects for wound severity (95 % CI [1.72 minutes, 8.17 minutes]), AI-based app use (95 % CI [1.90 minutes, 5.69 minutes]), and nurse qualification (95 % CI [0.51 minutes, 3.42 minutes]).

Given that the effect of app use was in the opposite direction of the original hypothesis, the variance exclusively attributable to app usage was not assessed by comparing the adjusted R2 values of the full model and the model without the app variable.

#### 3.2.2 Model for task load

No variables were found to have a statistically significant explanatory contribution to nurse task load.

#### 3.2.3 Model for guideline adherence

For the regression model predicting the dependent variable guideline adherence, the inclusion of the variable ward type resulted in an adjusted R² of 0.092, with an F-value of 9.806 (p = 0.002) and a power of 0.840.

The regression equation is:

Y=2.421+0.457×(WardType)

The standardized regression coefficient was 0.320 (p = 0.002, 95 % CI [0.117, 0.523]) for ward type. The variable was a statistically significant predictor, indicating a positive effect on guideline adherence.

The independence of residuals was confirmed. Despite violation of the homoscedasticity assumption, no further robust analyses were conducted, as the use of the AI-based app was not identified as a statistically significant predictor.

### 3.3 Usability

**Fig. 2:**
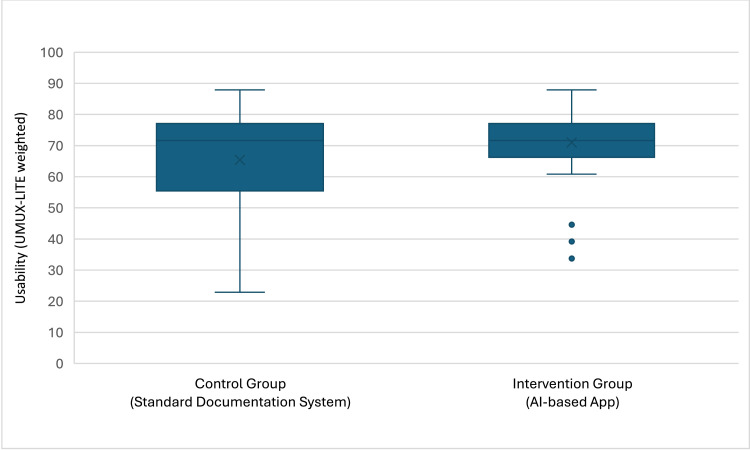
Boxplots of the usability (UMUX-LITE weighted) of the used wound documentation systems.

The mean usability score (UMUX-LITE weighted) of the standard documentation systems was 65.39 (median 71.65, Q1–Q3 = 55.40–77.07), and for the AI-based app it was 71.01 (median 71.65, Q1–Q3 = 66.23–77.07) (see Fig. 2).

The Mann–Whitney U test, applied due to variance heterogeneity between groups, revealed no statistically significant differences between the standard documentation systems and the AI-based app (*p* = 0.329), with a mean rank of 41.48 for the standard systems and 46.70 for the AI-based app.

### 3.4 AI model performance

The F1-score for predicting a pressure injury was 0.89, and for an incontinence-associated dermatitis it was 0.74. The micro F1-score calculated by aggregating true positives, false positives, and false negatives over all classes was 0.86.

The F1-scores for each wound category, as well as the micro F1-score across all wound categories of a wound type, are presented in Table 4.

**Table 4:** F1-Scores for wound classification.

	Wound Category	Positives	F1-Score
Pressure Injury (EPUAP^1^)	1	1	0.00
	2	18	0.70
	3^2^	12	0.29
	4	2	0.00
All Pressure Injury Categories			0.55
Incontinence-associated dermatitis (GLOBIAD^3^)	1A	4	0.57
	1B	0	0.00
	2A	5	0.00
	2B	3	-4
All Incontinence-associated Dermatitis Categories			0.29

1 European Pressure Ulcer Advisory Panel

2 Due to the datasets available for AI training, EPUAP pressure injury categories 5 and 6 were assigned to category 3.

3 Ghent Global IAD Categorization Tool

4 No F1-score reported due to no positive predictions for this class.

## 4 Discussion

To our knowledge, this study is the first to pilot an AI-based app supporting nursing wound management of pressure injury and incontinence-associated dermatitis in clinical care, investigating care duration, nurse task load, and guideline adherence. Contrary to the initial assumption of reduced care duration, an increase was observed, likely due to the lack of routine implementation and consequently limited staff experience, as well as more comprehensive documentation.

Previous research has demonstrated that AI-based tools can enhance workflow efficiency and resource allocation, primarily by optimizing human resource management rather than directly supporting patient care processes (Rosa et al., 2024). Bian et al. found that the implementation of an AI-based postoperative follow-up system saved nursing resources, though nurses were replaced rather than supported (Bian et al., 2020). Although nurses recognize AI’s potential to improve resource utilization (Almagharbeh, 2025), objective evidence of efficiency gains in direct care remains lacking. Our study specifically examined AI’s impact on care duration as a measure of direct care efficiency. However, no such gains were observed, as care times increased for the reasons discussed above.

Contrary to expectations, no statistically significant predictors of nurses’ task load were identified. Some participants found NASA-TLX items difficult to interpret, suggesting the need for alternative instruments or the weighted NASA-TLX in future research. Additionally, limited experience with the app and the observation setting may have influenced the reported task load. No comparable studies have assessed the AI’s impact on nursing task load in wound management. Kandaswamy et al. reported increased task load following AI-based sepsis prediction (Kandaswamy et al., 2025), whereas Zhang et al. found reduced cognitive but increased germane cognitive load using AI-assisted delirium prediction (Zhang et al., 2025). Unlike our study, Zhang et al. assessed only cognitive load rather than the overall task load, and in both studies, it was not measured immediately after the task. Moreover, task type, digital literacy, AI familiarity, and technical challenges may also affect perceived load (Almagharbeh, 2025).

In the linear regression analysis, ward type was the only statistically significant predictor of guideline adherence, with intensive care units showing higher adherence than other ward types. However, this finding may be biased by case distribution. Neither the use of the AI-based app nor other variables showed a statistically significant effect on guideline adherence. These findings suggest that contextual factors related to the ward environment, as well as the optional nature of certain AI-based app support features not utilized by all nursing staff, may affect adherence to clinical guidelines. Comparable studies on guideline adherence in the context of AI-based wound management tools are currently lacking. Zhang et al. reported an increase in adherence to an AI-generated care plan for delirium. However, no significant differences were observed regarding adherence to delirium assessment and risk factor assessment (Zhang et al., 2025). In contrast to our study, their analysis aggregated data across all patients cared for by a nurse or across an entire day for a single patient, whereas the measurement of guideline adherence in our study was based on a single data point.

The Mann–Whitney U test revealed statistically significant differences between the control and intervention groups in overall guideline adherence, blanch tests performance, and correct wound classification. Notably, a Hodges-Lehmann median difference was observed only for the overall guideline adherence and confidence intervals for all three measures included zero, indicating estimate uncertainty. No statistically significant differences were found in the wound type assessment, skin protection application, or pressure relief implementation.

One possible explanation is that the AI did not consistently classify wound type and classification accurately (see F1-scores), thereby limiting its support for precise wound assessment. Notably, despite a substantially higher F1-score for wound type compared to wound classification, the intervention group did not show a statistically significant improvement over the control group in wound type assessment. Conversely, the intervention group demonstrated statistically significantly better results in wound classification, even though the corresponding F1-scores were considerably lower. This suggests that additional information provided by the app, such as detailed guidance on wound classification, may have played a supportive role in enhancing performance in this domain. The observed differences in blanch test performance may be partially explained by the app workflow, which explicitly prompted users to enter the test results. Regarding other indicators of guideline adherence, it should be noted that the recommendations displayed by the app were based on wound characteristics documented by nursing staff and thus were not accurately presented when assessments were incorrect. Furthermore, the app’s function to display treatment recommendations was optional and not utilized by all nurses. Additionally, wound documentation was sometimes completed only after care had been provided, meaning that treatment recommendations were occasionally viewed post-intervention and therefore were no longer incorporated into care.

The AI-based app evaluated in this study received a slightly higher mean usability score (UMUX-LITE weighted) of 71.01 comparable to the standard documentation system, which had a mean of 65.39, although this difference was not statistically significant. The greater variability observed in the standard documentation systems, indicated by a higher standard deviation and a wider range, may reflect inconsistencies in user experience across different systems or clinical settings. The observed usability of the AI-based app may be attributed not only to the AI model itself but also to additional factors such as the use of a tablet instead of a digital camera, direct documentation on the tablet without requiring data transfer to another device and user familiarity with the app. These findings highlight the importance of tailoring digital tools to specific clinical environments to maximize acceptance and effectiveness, as well as implementing technical solutions that support and simplify processes, such as mobile devices for documentation.

The interpretability of the F1-scores in clinical practice is particularly limited for certain wound categories, such as pressure injury categories 1 and 4 as well as incontinence-associated dermatitis categories 1B and 2B, due to their uneven representation within the sample. Corresponding scores derived from test datasets with more balanced class distributions provide more representative performance metrics during model development (Brehmer et al., 2025). However, these metrics are less informative regarding the model’s performance in actual clinical practice. Several studies have evaluated various AI models for image-based prediction of wound types and pressure injury classification using test datasets. Notably, these models are generally not validated in actual clinical care environments (Reifs Jiménez et al., 2025). In contrast, Lau et al. investigated the accuracy of an AI-based application for classifying pressure injuries in clinical care, reporting an accuracy of 80 %. However, their sample included only 10 clinical images, and no details were provided on the distribution of wound categories within the sample (Lau et al., 2022). Meanwhile, Shiraishi et al. demonstrated that some conventional chatbots have now achieved good accuracy in classifying pressure injuries (Shiraishi et al., 2024).

## 5 Limitations

This study has several limitations that should be acknowledged. First, as a single-centre study, the generalizability of the findings is limited. Moreover, due to the sequential data collection, additional bias may have been introduced despite no formal changes to wound care processes, for example, through differences in workload or changes within the team. Additionally, there was a disproportionate number of observations from intensive care units compared to general wards. This imbalance may be attributable to factors such as a higher wound prevalence, greater willingness to participate, more available time, or the lower nurse-to-patient ratio in intensive care settings, which likely facilitates closer monitoring of skin changes by nursing staff. Moreover, the uneven prevalence of wound types and classifications in clinical practice led to an imbalanced distribution of these categories within the sample, potentially introducing bias into the results.

Regarding documentation duration, completeness of wound documentation was not systematically assessed for the standard documentation systems. Consequently, the longer documentation times observed in the intervention group may be explained by more thorough documentation within the AI-based app. Furthermore, these durations might have been affected by nursing staff’s limited experience and lack of routine use of the app.

Regarding nurse task load, some NASA-TLX items were reportedly challenging for nurses to interpret and answer precisely, and limited practical experience with the AI-based app may also have influenced these findings.

A key limitation in assessing guideline adherence was the absence of an official guideline for the management of incontinence associated dermatitis. As a result, the assessment had to rely solely on the available current literature, which meant that the included items remained relatively general for incontinence associated dermatitis. In addition, some items were more specific to pressure injury management or to the distinction between incontinence associated dermatitis and pressure injuries. Furthermore, only those aspects of care that could be directly observed during the wound care situation were captured. Elements of the care process that are not visually observable, such as the handling of excretions or the correct use of incontinence products, could therefore not be included in the assessment.

Furthermore, relevant factors potentially influencing guideline adherence and other outcomes could not be operationalized or included in the regression models. The large number of covariates required to capture all potential influences would have necessitated a substantially larger sample size, which was beyond the scope of this pilot study. The limited sample size may also have contributed to the non-significant results observed in several analyses. However, as a pilot study, the primary aim was feasibility and practical challenges, so the absence of statistically significant effects is not unexpected and does not diminish the value of the findings.

Lastly, the assessment of the AI system's performance via the F1-score is constrained by the imbalanced distribution of wound types and classifications, including the absence of certain classes within the sample. This limitation restricts the interpretability and generalizability of the metric. Given these constraints, as well as the low F1-scores observed for certain categories, the model in its current developmental stage is not suitable for autonomous classification. It should be employed exclusively as a decision-support tool and cannot substitute for expert clinical assessment, particularly for the less common categories. Nonetheless, inclusion of this performance metric was necessary, as the model’s performance directly impacts its potential to enhance nursing processes and support clinical decision making.

## 6. Conclusions

The pilot study showed that the AI-based wound management app was feasible in clinical care and may be associated with improvements in selected domains of guideline adherence, though estimates were uncertain and effects were not consistent across all indicators. No reductions in care duration or task load were observed. These findings indicate a limited immediate benefit for efficiency, while suggesting potential for supporting specific documentation and assessment tasks. Effective integration into practice will require routine use, training, and optimization of app function, and regulatory approval to ensure consistent clinical benefit.

Further multicentre studies are necessary to validate and generalize these findings. Future research should focus more on patient-centred outcomes, including wound healing progression, health-related quality of life, and patients’ perspectives and acceptance of AI-based wound management. Understanding how these tools affect the patient experience is crucial for successful integration into clinical practice.

Additionally, issues of cost-effectiveness need to be examined in more detail, particularly in light of the costs for devices, model adjustments, ensuring data storage and security, and the necessary training measures. At the same time, the potential cost savings resulting from AI implementation should be investigated further. Moreover, the broader impact of AI-based decision support on nursing practice requires comprehensive investigation, including its influence on clinical decision-making processes, professional identity, knowledge acquisition, and the nurse-patient relationship. Exploring these aspects will provide valuable insights into the acceptance and practical challenges of integrating AI into routine nursing care.

Research should also extend beyond inpatient settings to include other relevant care environments such as outpatient clinics, home care, and informal caregiving contexts. These settings present unique challenges and opportunities for the deployment of AI tools and may benefit from tailored functionalities. The integration of AI-based applications into digital wound care pathways, such as telenursing, also warrants further exploration.

Expanding the application’s scope to cover a wider range of data, wound types and incorporating advanced features, such as predictive modelling and detailed wound phase classification, could enhance clinical utility and support personalized care.

Robust effectiveness analyses, including randomized controlled trials and real-world evidence studies, are essential to establish the clinical benefits. Such evidence is critical for regulatory certification, reimbursement, and widespread implementation.

Securing funding for large-scale and long-term studies remains a pivotal challenge. Given the potential interest of healthcare payers in cost-effective, high-quality wound care solutions, collaborative efforts between academic institutions, industry partners, and healthcare providers will be necessary to advance research and support sustainable integration of AI into nursing practice.

## List of abbreviations

**Table:** 

AI	Artificial Intelligence
BCa	Bias-corrected and accelerated
CI	Confidence Interval
DKG	Deutsche Krankenhausgesellschaft (German Hospital Federation)
DNQP	Deutsches Netzwerk für Qualität in der Pflege (German Network for Quality Development in Nursing)
EPUAP	European Pressure Ulcer Advisory Panel
GLOBIAD	Ghent Global Incontinence-Associated Dermatitis Categorization Tool
ICD	International Classification of Diseases
LMU	Ludwig-Maximilians-Universität
NASA TLX	NASA Task Load Index
Q1–Q3	Interquartile Range
SD	Standard Deviation
UMUX-LITE	Usability Metric for User Experience Lite Version
VIF	Variance inflation factor

## 7 Declaration of generative AI and AI-assisted technologies in the writing process

During the preparation of this work, the authors used ChatGPT (GPT-5 mini) in order to improve readability. After using this tool, the authors reviewed and edited the content as needed and take full responsibility for the content of the publication.

## Funding

This work was supported by the German Federal Ministry of Research, Technology and Space (BMFTR) [grant number 16SV8813].

## Availability of data and materials

The datasets generated or analysed during the current study are not publicly available but are available from the study directors on reasonable request. The AI intervention and its codes are also not publicly accessible but can be obtained from the study directors on reasonable request.

## CRediT authorship contribution statement

**Hannah Pinnekamp:** Writing – original draft, Project administration, Methodology, Investigation, Formal analysis, Data curation, Conceptualization. **Vanessa Priester:** Writing – review & editing, Methodology, Investigation, Conceptualization. **Johanna Steidle:** Investigation. **Khalid Majjouti:** Writing – review & editing, Conceptualization. **Alexander Brehmer:** Writing – review & editing, Software, Data curation. **Michaela Tapp-Herrenbrück:** Writing – review & editing, Project administration. **Michael Aleithe:** Writing – review & editing, Software, Funding acquisition. **Jens Kleesiek:** Writing – review & editing, Funding acquisition. **Bernadette Hosters:** Writing – review & editing, Funding acquisition, Conceptualization. **Uli Fischer:** Writing – review & editing, Project administration, Methodology, Funding acquisition, Conceptualization.

## Declaration of competing interest

The authors declare the following financial interests/personal relationships which may be considered as potential competing interests:

Uli Fischer reports financial support was provided by German Federal Ministry of Research, Technology and Space (BMFTR). If there are other authors, they declare that they have no known competing financial interests or personal relationships that could have appeared to influence the work reported in this paper.

## Footnotes

1 German abbreviation for artificial intelligence

2 German term for pressure injury
